# Higher obstetric and perinatal risks for twins and the need for single embryo transfers in assisted reproduction

**DOI:** 10.1111/aogs.14639

**Published:** 2023-07-28

**Authors:** Kenny A. Rodriguez‐Wallberg, Zdravka Veleva

**Affiliations:** ^1^ Department of Reproductive Medicine Karolinska University Hospital, Stockholm, Sweden and Department of Oncology‐Pathology Karolinska Institutet Stockholm Sweden; ^2^ Department of Obstetrics and Gynecology University of Helsinki and Helsinki University Central Hospital Helsinki Finland

Twin pregnancies have a higher maternal and perinatal morbidity, when compared with singleton pregnancies. As most twin and higher‐order multiple pregnancies are the result of treatments using assisted reproductive technologies following the replacement of more than one embryo to the uterus, concerns have been raised and the development of good practice guidelines is ongoing. The European Society for Human Reproduction and Embryology, published their guideline on number of embryos to transfer in May 2023, and the guideline is currently under public stakeholder review.^1^

The data supporting higher pregnancy risks of twins vs singletons in the short‐term are substantial. According to a systematic review including 60 studies comparing both maternal and neonatal outcomes of twins vs singletons,^2^ the higher health risks identified in the mothers of twins included antenatal hospitalization, cesarean section, gestational diabetes, preterm labor, pregnancy‐induced hypertension, pre‐eclampsia, placental abruption, placenta previa, and postpartum hemorrhage. For the children, fetal and neonatal risks were also higher in twins than in singletons, and included congenital anomalies, preterm birth, low birthweight, admission to neonatal intensive care unit, perinatal mortality, and stillbirth.^2^ Moreover, the transfer of multiple embryos increases the risk of major congenital anomalies, small for gestational age, low birth weight, and preterm birth in singleton births.^3^ To date, studies with longer follow up have been lacking.

In this issue of *AOGS*, Wainstock et al.,^4^ report on the long‐term outcome, until 18 years of age, of 11 986 twins and 357 492 singletons in a population‐based cohort that included two main ethnic groups, Jewish and Arab‐Bedouin, both receiving standard obstetric health care in Israel. The researchers included only offspring that survived the pregnancy and delivery, so late miscarriages or stillbirths were not considered. Cases of congenital malformations or chromosomal abnormalities were also excluded. Although the mode of conception was not investigated, assisted reproductive technology treatments were involved in 24.1% of twin pregnancies vs 1.7% of singleton pregnancies.

The main findings of this study indicate that beyond the higher risks of preterm delivery and of being born by cesarean section of twin vs singleton pregnancies, twins still presented with higher risks of hospitalizations and childhood morbidity from birth and up to 18 years of age. Specific outcomes investigated included cardiac, respiratory, neurological, infectious dieseases, and malignancies. Multivariate analyses adjusted for maternal age, ethnicity, and offspring gender still indicated that twins, compared with singletons, were at increased risk for nearly all morbidities.

Our reflection in this editorial is about how much the medical community could do to reduce the risks for mothers and children. If single embryo transfers become the standard, several negative outcomes associated with prematurity that have an impact in the health of the children might be reduced.^1^ Additional risks associated with multiple embryo transfers include also higher risks for ectopic pregnancy, which also increases with the number of embryos transferred.^5^ Finally, singleton pregnancies that develop after double embryo transfers have been associated with a higher risk of neonatal death and low birthweight compared with singleton pregnancies resulting from single embryo transfers in a recent Nordic population‐based study.^6^
